# Characterization of *trans*-3-Methylglutaconyl CoA-Dependent Protein Acylation

**DOI:** 10.3390/metabo13070862

**Published:** 2023-07-20

**Authors:** Elizabeth A. Jennings, Edward Cao, Irina Romenskaia, Robert O. Ryan

**Affiliations:** Department of Biochemistry & Molecular Biology, University of Nevada, Reno, NV 89557, USA; elizabethjennings@nevada.unr.edu (E.A.J.); ecao@nevada.unr.edu (E.C.); irina@unr.edu (I.R.)

**Keywords:** 3MGC aciduria, protein 3MGCylation, acetyl CoA diversion, immunoblot, inborn error of metabolism

## Abstract

3-methylglutaconyl (3MGC) CoA hydratase (AUH) is the leucine catabolism pathway enzyme that catalyzes the hydration of *trans*-3MGC CoA to 3-hydroxy, 3-methylglutaryl (HMG) CoA. In several inborn errors of metabolism (IEM), however, metabolic dysfunction can drive this reaction in the opposite direction (the dehydration of HMG CoA). The recent discovery that *trans*-3MGC CoA is inherently unstable and prone to a series of non-enzymatic chemical reactions provides an explanation for 3MGC aciduria observed in these IEMs. Under physiological conditions, *trans*-3MGC CoA can isomerize to *cis*-3MGC CoA, which is structurally poised to undergo intramolecular cyclization with the loss of CoA, generating *cis*-3MGC anhydride. The anhydride is reactive and has two potential fates; (a) hydrolysis to yield *cis*-3MGC acid or (b) a reaction with lysine side-chain amino groups to 3MGCylate substrate proteins. An antibody elicited against a 3MGC hapten was employed to investigate protein acylation in incubations containing recombinant AUH, HMG CoA, and bovine serum albumin (BSA). The data obtained show that, as AUH dehydrates HMG CoA to *trans*-3MGC CoA, BSA is acylated. Moreover, α-3MGC IgG immunoblot signal intensity correlates with AUH concentration, HMG CoA substrate concentration, and incubation time. Thus, protein 3MGCylation may contribute to the phenotypic features associated with IEMs that manifest 3MGC aciduria.

## 1. Introduction

**Figure 1:**
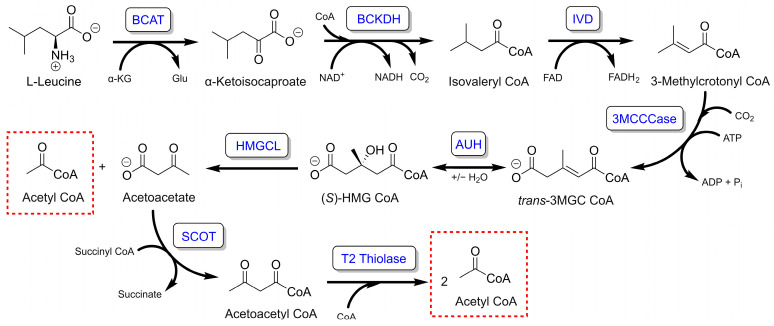
Leucine degradation pathway in muscle tissue mitochondria. At completion, this pathway yields three acetyl CoA per leucine residue. BCAT = branched-chain aminotransferase; BCKDH = branched-chain α-keto acid dehydrogenase; IVD = isovaleryl CoA dehydrogenase; 3MCCCase = 3-methylcrotonyl CoA carboxylase; AUH = 3-methylglutaconyl CoA hydratase; HMGCL = HMG CoA lyase; SCOT = succinyl CoA:3-oxoacid CoA transferase. Pathway product acetyl CoA moieties are depicted in hatched red boxes.

Isoleucine, valine, and leucine comprise the three branched-chain amino acids. Collectively, these amino acids represent an important source of energy, especially in muscle tissue. The catabolism of leucine to energy yielding metabolites is initiated by transamination to remove the amino group followed by decarboxylation-dependent conversion to isovaleryl CoA (Figure 1). Subsequent dehydrogenation of isovaleryl CoA yields the α–ß unsaturated acyl CoA, 3-methylcrotonyl CoA. The methyl group on the ß-carbon of 3-methylcrotonyl CoA prevents oxidation of this carbon to a ketone. As such, the usual sequence of hydration followed by ß-carbon oxidation is not possible. Instead, a biotin-dependent carboxylation reaction adds a carboxyl moiety to the acyl chain, forming the terminally carboxylated, α–ß unsaturated acyl CoA, *trans*-3-methylglutaconyl (3MGC) CoA. This ATP-dependent reaction is catalyzed by 3-methylcrotonyl CoA carboxylase. Subsequently, *trans*-3MGC CoA is hydrated by 3MGC CoA hydratase (AUH) to form 3-hydroxy, 3-methylglutaryl (HMG) CoA, which is cleaved by HMG CoA lyase to yield acetyl CoA and acetoacetate. In extrahepatic tissues, succinyl CoA-oxoacid CoA transferase converts acetoacetate to acetoacetyl CoA, which is subject to thiolytic cleavage by acetoacetyl CoA thiolase, yielding two acetyl CoA. Thus, each leucine molecule yields three acetyl CoAs that can enter the TCA cycle to produce ATP. 

**Figure 2:**
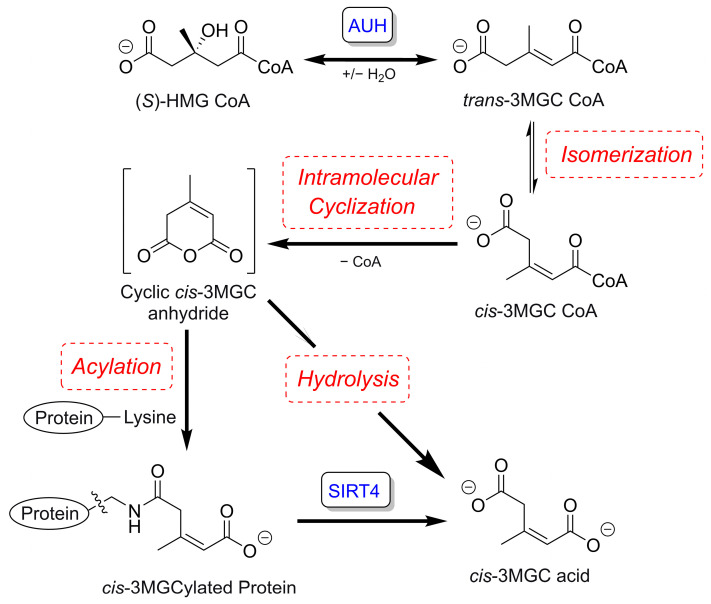
Non-enzymatic chemical reaction scheme from *trans*-3MGC CoA. When hydration of *trans*-3MGC CoA is prevented by an inborn error of metabolism in *AUH*, for example, this metabolite is subject to a series of non-enzymatic chemical reactions including isomerization to *cis*-3MGC CoA, intramolecular cyclization to *cis*-3MGC anhydride plus CoA, and hydrolytic cleavage of the cyclic anhydride to yield the organic acid, *cis*-3MGC acid, which is excreted in urine. In addition to this outcome, *cis*-3MGC anhydride can react with protein lysine side-chain amino groups to covalently 3MGCylate these residues. 3MGCylated proteins may be deacylated by the NAD^+^ requiring enzyme sirtuin 4 (SIRT4), yielding *cis*-3MGC acid as a product. Non-enzymatic chemical reactions in this process are depicted in hatched red boxes.

Interest in the pathway intermediate, *trans*-3MGC CoA, has increased with the knowledge that inborn errors of metabolism (IEM) which affect AUH, encoded by the *AUH* gene or HMG CoA lyase (encoded by *HMGCL*), result in urinary excretion of large quantities of 3MGC acid [1]. In these “primary” 3MGC acidurias, leucine catabolism is blocked due to an inability to either hydrate *trans*-3MGC CoA (AUH deficiency) or cleave HMG CoA into acetoacetate and acetyl CoA (HMGCL deficiency). In these enzyme deficiencies, as *trans*-3MGC CoA accumulates, it is subject to three sequential non-enzymatic chemical reactions, generating the organic acid, *cis*-3MGC acid (Figure 2), which is excreted in urine (3MGC aciduria). These reactions include: (1) Isomerization of *trans*-3MGC CoA to *cis*-3MGC CoA; (2) intramolecular cyclization to *cis*-3MGC anhydride and free CoA; and (3) hydrolysis of the anhydride to yield *cis*-3MGC acid [1]. This reaction sequence provides an explanation for the massive excretion of 3MGC acid in subjects harboring these enzyme deficiencies [2,3]. Another interesting and important aspect of this reaction sequence is that 3MGC anhydride is susceptible to nucleophilic attack by protein lysine side-chain amino groups to form a covalent acyl linkage. Thus, in addition to anhydride hydrolysis, protein 3MGCylation represents an alternate fate of *trans*-3MGC CoA. Detection of 3MGCylated proteins can be achieved via immunoblot analysis using an α-3MGC IgG [4].

In a distinct group of ~20 IEMs that affect mitochondrial energy metabolism (termed “secondary” 3MGC acidurias), no leucine catabolism pathway enzyme deficiencies exist. These IEMs affect genes that encode disparate mitochondrial proteins [5]. This result is surprising in that, aside from leucine catabolism, 3MGC CoA appears nowhere else in human intermediary metabolism. When considering the various gene mutations that lead to secondary 3MGC aciduria, a previously unknown metabolic route to *trans*-3MGC CoA has been proposed. The “acetyl CoA diversion pathway” is initiated by IEMs in genes that participate, directly or indirectly, in aerobic energy metabolism [1]. In this process, mutations that adversely affect electron transport chain function result in inhibition of the TCA cycle. When this occurs, acetyl CoA is unable to enter the cycle and, instead, is diverted to 3MGC CoA in three enzyme-mediated steps. These include (a) condensation of two acetyl CoA to form acetoacetyl CoA, (b) condensation of acetoacetyl CoA with acetyl CoA to yield HMG CoA, and (c) dehydration of HMG CoA to form *trans*-3MGC CoA [6]. Once formed by this route, *trans*-3MGC CoA cannot proceed further up the leucine degradation pathway because the next reaction, catalyzed by 3-methylcrotonyl CoA carboxylase, is irreversible. Under these conditions, *trans*-3MGC CoA undergoes non-enzymatic chemical reactions that yield *cis*-3MGC anhydride (see Figure 2), ultimately yielding 3MGC acid and 3MGCylated proteins.

In the present report, AUH-mediated dehydration of HMG CoA was investigated by measuring protein 3MGCylation in incubations containing isolated recombinant AUH, HMG CoA, and bovine serum albumin (BSA). The results obtained reveal a positive correlation between the extent of BSA 3MGCylation and various reaction parameters, including enzyme and substrate concentrations, and incubation time. Studies of the effect of reaction temperature on BSA 3MGCylation provide support for the non-enzymatic nature of the terminal reaction sequence.

## 2. Materials and Methods

### 2.1. Materials

(*R*,*S*)-HMG CoA and BSA were obtained from Millipore-Sigma and used without further modification. Recombinant *A. thaliana* AUH was expressed in *E. coli* and isolated as described previously [7]. Rabbit α-3MGC IgG was prepared as described [4].

### 2.2. AUH Activity Assay

As specified in the text, assays were conducted in one of two buffers, “Tris buffer” (100 mM Tris HCl, pH 8.0, 10 mM EDTA) or “HEPES buffer” (25 mM HEPES, pH 8.0, 75 mM NaCl, 10 mM EDTA). Assays (100 µL total volume) were initiated by the addition of the enzyme to samples containing specified amounts of HMG CoA and BSA (0.5 mg/mL) followed by incubation at 37 °C for indicated times. Following incubation, aliquots of assay mixtures were subjected to immunoblot analysis to measure AUH-dependent dehydration of HMG CoA to *trans*-3MGC CoA and the subsequent 3MGCylation of BSA. 

### 2.3. Immunoblot Analysis

Samples were applied to wells of a 4–20% acrylamide gradient gel. Proteins were separated by SDS-PAGE and transferred to a PVDF membrane. To determine the extent of BSA 3MGCylation, the membrane was blocked in 5% milk powder in Tris buffered saline containing 0.05% Tween 20 (TTBS) and incubated with rabbit α-3MGC IgG (1:40,000) for 16 h at 4 °C. The membrane was then washed with TTBS and incubated with goat α-rabbit IgG HRP-conjugated secondary antibody (1:4000 dilution) for 1 h at room temperature. The membrane was then washed and antibody binding was detected via chemiluminescence using SuperSignal West Pico PLUS chemiluminescent substrate (Thermo Scientific, Waltham, MA, USA). Densitometric analysis was performed using tools native to Image Lab software version 6.0.1 (Bio-Rad, Hercules, CA, USA). Relative band intensities were quantified by normalizing against the most intense band on the same blot.

### 2.4. Optimization of Enzyme and Substrate Concentrations

Experiments were conducted to identify optimal conditions for detection of AUH-dependent 3MGCylation of BSA. To define an optimal enzyme concentration, increasing amounts of AUH were added to assay mixtures containing 250 µM HMG CoA and 0.5 mg/mL BSA, followed by 24 h incubation at 37 °C in Tris buffer. In subsequent experiments, designed to determine an optimal concentration of HMG CoA substrate, assay mixtures containing 1 µg AUH and 0.5 mg/mL BSA were incubated with a specified range of HMG CoA concentrations for 24 h at 37 °C in Tris buffer. Immunoblot analysis was then performed to determine the extent of AUH-dependent BSA 3MGCylation. 

### 2.5. Effect of Buffer Composition on AUH-Dependent 3MGCylation of BSA

Parallel reactions were conducted in assay mixtures containing 0.5 mg/mL BSA and 200 µM HMG CoA in either Tris buffer or HEPES buffer. Assays were initiated by adding 0.01 µg/µL AUH followed by incubation at 37 °C for 24 h. AUH assays were also conducted in Tris buffer and HEPES buffer supplemented with 100 mM glycine. Following incubation, aliquots from each assay mix were subjected to α-3MGC IgG immunoblot analysis. 

### 2.6. Effect of Buffer Ionic Strength on AUH-Dependent BSA 3MGCylation

AUH assays were conducted in HEPES buffer containing BSA (0.5 mg/mL), HMG CoA (200 µM), and AUH (0.01 µg/µL). The NaCl content in the assay buffer ranged from 0, 25, 75, 150, 300, 500, and 1000 mM, respectively. Assays were initiated by introduction of AUH followed by incubation at 37 °C for 24 h. Following incubation, α-3MGC IgG immunoblot analysis was performed.

### 2.7. Effect of Incubation Time on AUH-Mediated BSA 3MGCylation 

To determine the effect of incubation time on AUH activity, assays were conducted using optimized enzyme and substrate concentrations (0.01 µg/µL AUH and 200 µM HMG CoA) in HEPES buffer containing 0.5 mg/mL BSA. Samples were incubated at 37 °C for 10, 12, 14, 16, 18, 20, 22, and 24 h, respectively. Following incubation, aliquots of each reaction mixture were subjected to α-3MGC IgG immunoblot analysis. 

### 2.8. Effect of Incubation Temperature on AUH-Dependent BSA 3MGCylation

To determine the effect of incubation temperature on AUH activity, assays were conducted using optimized enzyme and substrate concentrations (0.01 µg/µL AUH and 200 µM HMG CoA). Reactions were conducted in HEPES buffer containing 0.5 mg/mL BSA at the following temperatures (4, 25, 37, 50, 60, and 70 °C, respectively) for 24 h. Following incubation, aliquots of reaction mixtures were subjected to α-3MGC IgG immunoblot analysis. 

### 2.9. Effect of AUH Preincubation Conditions on BSA 3MGCylation

Samples of AUH (2.5 µg) were pre-incubated for 15 min at 37, 50, 60, 70, or 80 °C. Following pre-incubation, the samples were cooled and used in assays to detect AUH-dependent 3MGCylation of BSA. Assays were conducted in HEPES buffer containing 0.5 mg/mL BSA, 200 µM HMG CoA, and 0.01 µg/µL pre-incubated AUH at 37 °C for 24 h. Following incubation, aliquots of each assay mixture were subjected to α-3MGC IgG immunoblot analysis. 

### 2.10. Liquid Chromatography—Mass Spectrometry Analysis 

An incubation containing AUH (0.01 µg/µL), HMG CoA (200 µM), and BSA (0.5 mg/mL) was conducted in HEPES buffer at 37 °C for 24 h. Following incubation, the sample was digested with trypsin and subjected to liquid chromatography-mass spectrometry analysis on an Orbitrap Eclipse mass spectrometer (Thermo Scientific, Waltham, MA, USA), essentially as described elsewhere [8]. Two control incubations, containing AUH and BSA only or HMG CoA and BSA only, were processed and analyzed in the same way as the test sample. Data analysis was performed using Sequest version v.27, rev. 11 (Thermo Scientific, Waltham, MA, USA) and Scaffold Q^+^ (Proteome Software, Portland, OR, USA).

## 3. Results

### 3.1. Effect of AUH Concentration on HMG CoA-Dependent BSA 3MGCylation 

**Figure 3:**
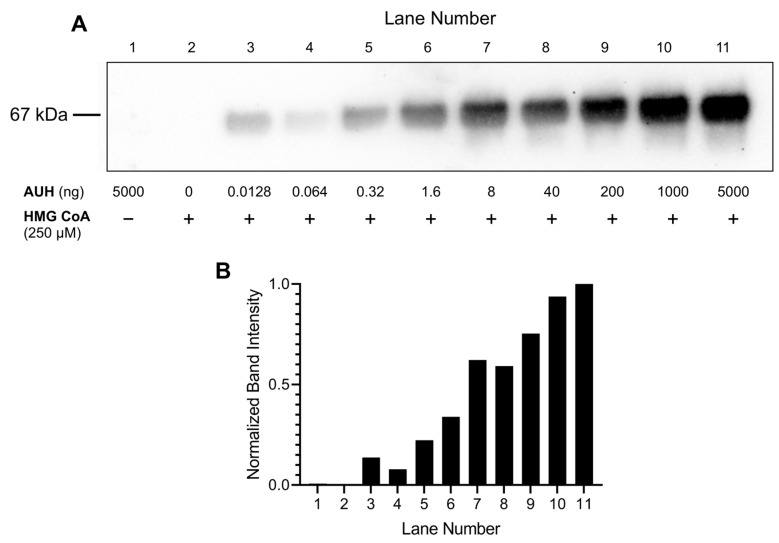
Effect of AUH concentration on the formation of 3MGCylated BSA from HMG CoA. (**A**) AUH activity assays were conducted by incubating specified amounts of AUH with 250 µM HMG CoA and 50 µg BSA in 100 mM Tris HCl, pH 8.0, at 37 °C for 24 h (100 µL final volume). Following incubation, aliquots of each assay mixture were subjected to SDS-PAGE and transferred to a PVDF membrane. After blocking non-specific sites and washing, the membrane was probed with α-3MGC IgG and developed as described in Material and Methods. (**B**) Densitometric analysis of the immunoblot from Panel A was conducted to quantify relative band intensities. Results presented are representative of an experiment that was performed on two separate occasions.

Initial assays contained a fixed amount of HMG CoA (250 µM) and BSA (0.5 mg/mL) and variable amounts of AUH. Following incubation at 37 °C for 24 h, the extent of BSA 3MGCylation was assessed. The results presented in Figure 3A revealed that, in the absence of HMG CoA or AUH, no BSA 3MGCylation occurred. By contrast, at AUH concentrations ranging from 12.8 pg to 5 µg (per 100 µL assay), BSA 3MGCylation signal intensity steadily increased. Densitometric analysis of immunoblot band intensity confirmed a positive correlation between AUH concentration and the extent of BSA 3MGCylation (Figure 3B). Based on these results, 1 µg AUH per assay (100 µL total volume) was employed in all subsequent experiments.

### 3.2. Effect of Substrate Concentration on AUH-Dependent BSA 3MGCylation

**Figure 4:**
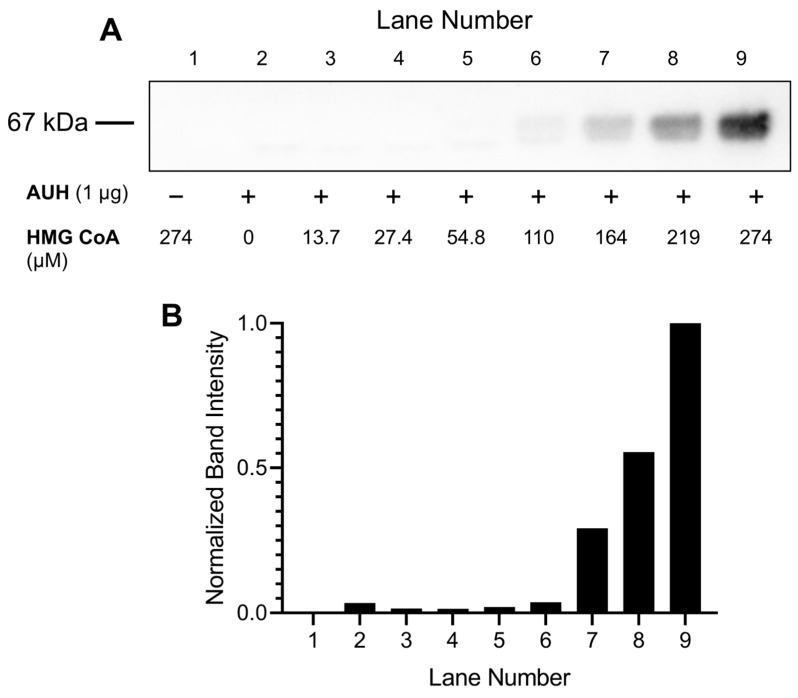
Effect of HMG CoA substrate concentration on AUH-dependent 3MGCylation of BSA. (**A**) AUH activity assays (100 µL final volume) were conducted by incubating AUH (1 µg) and BSA (50 µg) and indicated amounts of HMG CoA in Tris buffer at 37 °C for 24 h. Following incubation, an aliquot of each assay mixture was subjected to SDS-PAGE and transferred to a PVDF membrane. After blocking non-specific sites and washing, the membrane was probed with α-3MGC IgG and developed. (**B**) Densitometric analysis of the immunoblot from Panel A was conducted to quantify relative band intensities. The results presented are representative of an experiment that was performed on two separate occasions.

HMG CoA serves as substrate for a reversible, AUH-dependent, dehydration reaction that generates *trans*-3MGC CoA. To determine the effect of HMG CoA concentration on the amount of 3MGCylated BSA formed, a fixed amount of AUH (0.01 µg/µL) and BSA (0.5 mg/mL) was incubated with increasing amounts of HMG CoA (14 µM–274 µM). When either HMG CoA or AUH were omitted from incubations, no BSA 3MGCylation was detected (Figure 4A). On the other hand, in incubations containing HMG CoA, AUH, and BSA, a positive correlation between HMG CoA concentration and 3MGCylated BSA signal intensity was observed. This trend was confirmed by densitometric analysis of immunoblot lanes (Figure 4B). Based on the relative immunoblot signal intensity under different conditions, 200 µM HMG CoA was used in all subsequent assays.

### 3.3. The Effect of Buffer Composition on AUH-Dependent BSA 3MGCylation

**Figure 5:**
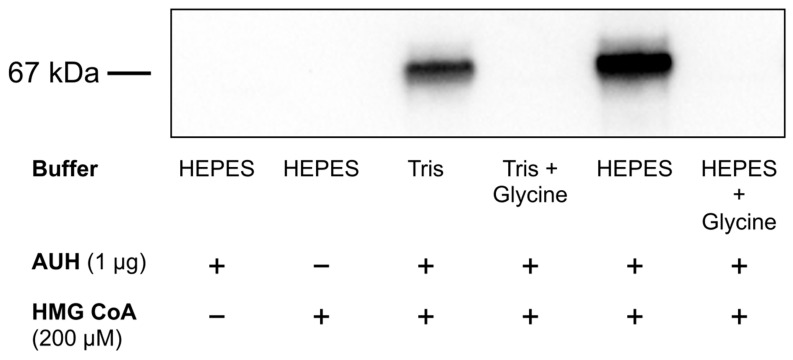
Effect of buffer composition on AUH- and HMG CoA-dependent 3MGCylation of BSA. AUH activity assays were conducted in Tris buffer or HEPES buffer (100 µL final volume). Where indicated, 100 mM glycine was included in incubations. Reactions containing AUH (1 µg), HMG CoA (200 µM), and BSA (50 µg) were incubated for 24 h at 37 °C. Following incubation, an aliquot of each assay mixture was subjected to SDS-PAGE and transferred to a PVDF membrane. After blocking non-specific sites and washing, the membrane was probed with α-3MGC IgG and developed. Results presented are representative of an experiment that was performed on four separate occasions.

Previous studies investigating AUH activity employed Tris as the buffering agent [9,10,11,12]. However, knowledge that *trans*-3MGC CoA is susceptible to isomerization/cyclic anhydride formation suggests that *cis*-3MGC anhydride may react with the free amino group on Tris to yield 3MGCylated Tris as a product and, thereby, attenuate 3MGCylated BSA signal intensity. Compared to AUH assays conducted in Tris buffer, parallel assays conducted in HEPES buffer gave rise to enhanced 3MGCylated BSA signal intensity (Figure 5). Given that 3MGCylated BSA was detected despite the presence of 100 mM Tris HCl, it was hypothesized that the primary amine in Tris does not serve as an optimal acylation target of 3MGC anhydride. To examine this further, the ability of glycine to inhibit AUH-dependent BSA 3MGCylation was examined. Whether AUH assays were conducted in Tris or HEPES buffer, when 100 mM glycine was included, no BSA 3MGCylation was detected. From this point on, all AUH assays were conducted in HEPES buffer.

### 3.4. Effect of Ionic Strength on BSA 3MGCylation

To determine the effect of solution ionic strength on AUH activity, assays were conducted in 25 mM HEPES, pH 8.0, supplemented with increasing amounts of NaCl. These assays employed standard conditions (1 µg AUH, 200 µM HMG CoA, and 50 µg BSA; 100 µL final volume) and were incubated at 37 °C for 24 h. Following incubation, the extent of BSA 3MGCylation was assessed via immunoblot analysis. In assays containing NaCl at concentrations ranging from 0 to 1 M, 3MGCylated BSA signal intensities were equivalent. 

### 3.5. Effect of Incubation Time on AUH-Dependent BSA 3MGCylation

**Figure 6:**
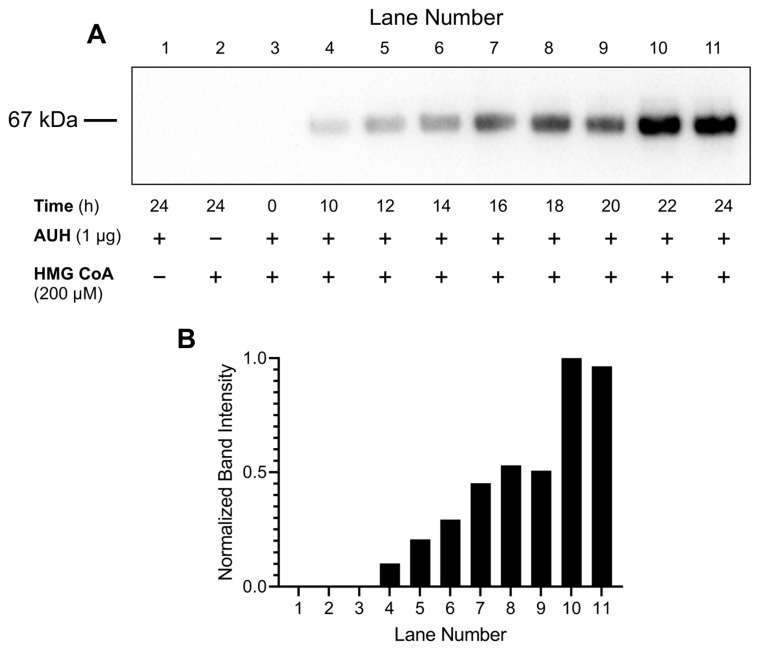
Effect of incubation time on AUH- and HMG CoA-dependent 3MGCylation of BSA. (**A**) AUH activity assays were conducted in HEPES buffer (100 µL final volume) containing AUH (1 µg), HMG CoA (200 µM), and BSA (50 µg) at 37 °C for specified times intervals. Following incubation, an aliquot of each assay mixture was subjected to SDS-PAGE and transferred to a PVDF membrane. After blocking non-specific sites and washing, the membrane was probed with α-3MGC IgG and developed. (**B**) Densitometric analysis of the immunoblot from Figure 4A was conducted to quantify relative band intensities. The results presented are representative of an experiment that was performed on four separate occasions.

AUH assays were conducted to evaluate the effect of incubation time on 3MGCylated BSA signal intensity. Under these conditions, no 3MGCylated BSA was detected at 0 h (Figure 6A). After 10 h incubation, however, a distinct band of 3MGCylated BSA was observed, which increased in intensity as a function of incubation time between 10 h and 24 h. At 24 h, a strong band corresponding to 3MGCylated BSA, was detected. Densitometric analysis of immunoblot band intensity (Figure 6B) revealed a positive correlation between incubation time and BSA 3MGCylation. 

### 3.6. Effect of Incubation Temperature on AUH-Dependent BSA 3MGCylation

**Figure 7:**
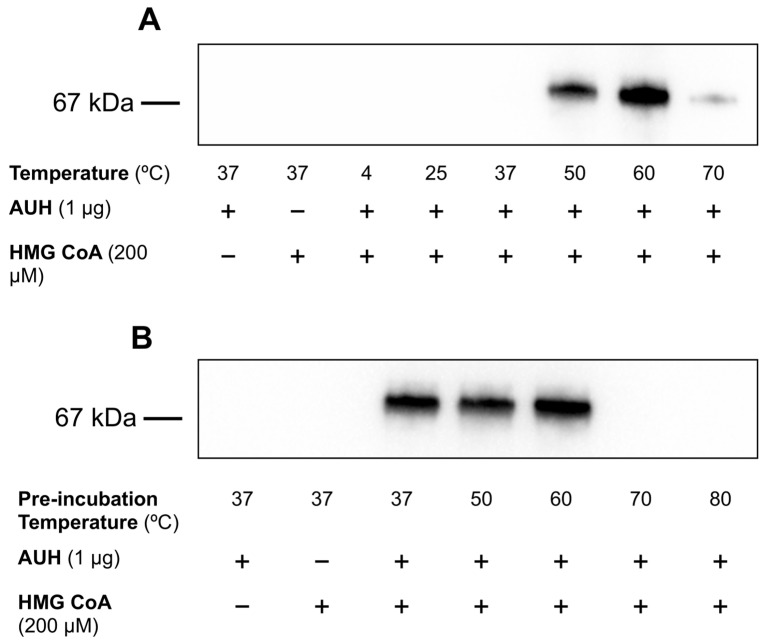
Effect of temperature on AUH- and HMG CoA-dependent 3MGCylation of BSA. (**A**) AUH activity assays (100 µL final volume) were conducted in HEPES buffer containing AUH (1 µg), HMG CoA (200 µM), and BSA (50 µg) for 24 h at the indicated temperatures. Following incubation, an aliquot of each assay mixture was subjected to SDS-PAGE and transferred to a PVDF membrane. After blocking non-specific sites and washing, the membrane was probed with α-3MGC IgG and developed. Results presented are representative of an experiment that was performed on four separate occasions. (**B**) AUH (2.5 µg) was pre-incubated at the indicated temperatures for 15 min prior to use in assays of BSA 3MGCylation. Following pre-incubation, 1 µg aliquots of AUH were introduced into assay mixtures containing HEPES buffer, 200 µM HMG CoA and 50 µg BSA and incubated for 24 h at 37 °C. An aliquot of each assay mixture was then subjected to SDS-PAGE and transferred to a PVDF membrane. After blocking non-specific sites and washing, the membrane was probed with α-3MGC IgG and developed. Results presented are representative of an experiment that was performed on four separate occasions.

In subsequent assays, the effect of incubation temperature on AUH-dependent BSA 3MGCylation was examined following 24 h incubations. In the absence of HMG CoA, or AUH, no 3MGCylated BSA was detected (Figure 7A). Likewise, in assays conducted at 4 °C in the presence of HMG CoA and AUH, no BSA 3MGCylation occurred. Between 37 °C and 60 °C, however, when HMG CoA and AUH were present, increased BSA 3MGCylation was detected. At 70 °C and above, however, BSA 3MGCylation signal intensity was strongly reduced. It is noteworthy that, whereas the assay conducted at 37 °C with AUH, HMG CoA and BSA present gave rise to a very faint BSA 3MGCylation signal, this is due to the much stronger signal intensity observed in assays conducted at 60 °C, which required a lower exposure time. At higher exposure times, where the 60 °C lane is strongly overexposed, the signal intensity for the 37 °C sample was similar to results shown in Figure 3, Figure 4 and Figure 5. In another experiment, AUH was subjected to a 15 min pre-incubation at 37, 50, 60, 70, and 80 °C. Following this, an aliquot of each pre-incubated AUH sample was added to assay mixtures containing 200 µM HMG CoA and 0.5 mg/mL BSA and incubated at 37 °C for 24 h in HEPES buffer. Immunoblot analysis of the reaction mixtures revealed that, under these conditions, pre-incubation of AUH at 37, 50, or 60 °C yielded similar BSA 3MGCylation signal intensities (Figure 7B) while little or no evidence of BSA 3MGCylation was detected when AUH was preincubated for 15 min at 70 or 80 °C.

### 3.7. Mass Spectrometry Analysis of BSA Acylation

Mass spectrometry analysis of the reaction products obtained following incubation of AUH, HMG CoA, and BSA was performed to confirm that 3MGCylation occurred. The results obtained show that lysine residues in BSA were covalently modified by 3MGC moieties (126.03 Da molecular weight increment), consistent with immunoblot analysis reported herein. In addition to 3MGCylation, however, the HMGylation of BSA lysine residues (144.04 Da molecular weight increment), was also detected. This occurs because HMG CoA (used as the substrate in AUH assay mixtures) is also capable of acylating lysine residues [13]. Importantly, the antibody used to detect 3MGCylation does not recognize HMGylated proteins [12]. Thus, there is competition between HMGylation and 3MGCylation for lysine residues on BSA. When HMG CoA, AUH, and BSA were incubated, following trypsin digestion and liquid chromatography—mass spectrometry analysis, evidence for significant HMGylation (detected on 39 of the 54 covered lysine residues; 72.2%). Likewise, 3MGCylation was detected on 30 of the 54 covered lysine residues (55.6%). Based on these results it is evident that, in this assay system, HMG CoA and *trans*-3MGC CoA compete for common acylation sites on BSA, in all likelihood the most solvent exposed lysine residues. When AUH and BSA were incubated (no HMG CoA), 3MGCylation was not detected (0 of 36 covered lysines), while 1 HMGylated lysine residue was detected among the 36 covered lysines (2.8%) in this sample. When HMG CoA and BSA (no AUH) were incubated, HMGylation was detected on 39 of 49 covered lysine residues (79.6%). In this sample, 3MGCylation was detected on 2 of 49 covered lysine residues (4.1%). This result, as well as the detection of 1 HMGylated residue in incubations lacking HMG CoA, is ascribed to minor sample contamination (carry over) between runs since the heavily acylated sample was run first during this analysis.

## 4. Discussion

Evidence suggests 3MGC acid is toxic and induces oxidative stress in brain tissue [14]. As such, individuals with inborn errors of metabolism characterized by deficiencies in AUH or HMGCL experience lifelong neurological symptoms, including muscle coordination impairment and febrile seizures. Whereas the accumulation of 3MGC acid is proposed to contribute to these neurological symptoms, it is now recognized that the biochemical pathway to 3MGC acid has a branch point that leads to non-specific protein 3MGCylation. Although the NAD^+^-dependent deacylase, sirtuin 4, is able to remove 3MGC moieties from proteins [15], the efficiency with which this occurs is unknown. Thus, it is conceivable that protein 3MGCylation contributes to the pathological consequences associated with these inborn errors of metabolism. 

AUH assay methods reported previously [9,10,11] generally involve measurement of AUH-dependent dehydration of HMG CoA to *trans*-3MGC CoA. Given the recent finding that *trans*-3MGC CoA is unstable and undergoes a series of non-enzymatic chemical reactions to yield 3MGC acid or 3MGCylated proteins, the fidelity of these assays may be questioned [12,16]. In previous studies of 3MGC aciduria, an antibody specific for 3MGC moieties was generated [4]. This antibody was shown to bind specifically to 3MGCylated proteins, providing the basis for immunodetection of AUH reaction products. The concept addressed in the present study is that, once 3MGC CoA accumulates, either because of a deficiency in AUH or HMGCL (primary 3MGC aciduria) or via the acetyl CoA diversion pathway (secondary 3MGC aciduria), some portion of this metabolite pool isomerizes to *cis*-3MGC CoA and undergoes intramolecular cyclization to 3MGC anhydride plus free CoA. Once formed, the anhydride has two possible fates, including hydrolysis to yield *cis*-3MGC acid or reaction with protein lysine side-chain amino groups to form a covalent adduct (i.e., protein 3MGCylation). Mass spectrometry analysis of BSA following incubation with AUH and HMG CoA confirmed that 3MGCylation occurred and is responsible for the immunoblot signal detected.

To further explore *trans*-3MGC CoA-dependent protein 3MGCylation, in vitro experiments were conducted. Standard assay conditions were established with respect to AUH concentration, HMG CoA substrate concentration, incubation time, and buffer composition. In studies of this latter parameter, a Tris buffer was initially employed. In subsequent studies, however, it was determined that, under identical conditions, HEPES buffer gave rise to a 3MGCylated BSA immunoblot signal intensity that was noticeably stronger than parallel assays conducted in Tris buffer. In considering the molecular basis for this result, it was hypothesized that the free amino group in Tris functions as an alternate acylation target of 3MGC anhydride. A curious aspect of this concept, however, is the fact that, although Tris was present at 100 mM, BSA 3MGCylation was still observed. To explore this further, experiments were performed by including glycine (100 mM) in assays conducted in Tris buffer or HEPES buffer. In both cases, compared to control assays, inclusion of glycine resulted in complete inhibition of BSA 3MGCylation. Based on these data, it may be concluded that the three hydroxymethyl moieties in Tris reduce its reactivity toward 3MGC anhydride, by steric hindrance or otherwise, such that it functions as a relatively poor competitive inhibitor of BSA 3MGCylation. 

Interesting results were also obtained when the effect of reaction temperature on AUH-dependent BSA 3MGCylation was examined. In these experiments, assays were conducted at various temperatures to determine the optimal incubation temperature for BSA 3MGCylation. Unexpectedly, a steady increase in BSA 3MGCylation signal was observed as the incubation temperature increased from 37 °C to 60 °C. Above this temperature (i.e., 70 °C) the signal for BSA 3MGCylation was significantly reduced. Based on this result, it was hypothesized that decreased BSA 3MGCylation observed in incubations conducted at 70 °C is caused by AUH denaturation, while the enzyme remained stable at 60 °C. If true, then the large increase in BSA 3MGCylation signal intensity observed at 60 °C versus 37 °C, for example, may be attributed to an increase in the rate of non-enzymatic chemical reactions by the dehydration product, *trans*-3MGC CoA. If these reactions proceed at a faster rate at increased temperature, then an enhanced signal for protein 3MGCylation may be expected. This interpretation, however, is complicated by the fact that the rate of AUH-mediated dehydration of HMG CoA is also expected to increase as a function of increasing temperature, although it is anticipated that the reverse reaction (hydration of *trans*-3MGC CoA) will also be increased. Another possibility is that the relative proportion of *cis*-3MGC anhydride hydrolysis versus acylation is affected by temperature. Another consideration is that, at elevated temperature, lysine side-chain amino groups on BSA become more exposed, increasing their accessibility to 3MGCylation by the anhydride. Further experiments are required to decipher the chemical basis for the large increase in AUH-dependent 3MGCylation at 60 °C versus 37 °C.

## 5. Conclusions

The present study provides evidence that protein acylation mediated by the AUH reaction product, *trans*-3MGC CoA, is dependent on several factors. When BSA is employed as an acylation acceptor, the amount of BSA 3MGCylation observed is directly related to assay composition and incubation conditions. The results obtained support the concept that *trans*-3MGC CoA has unique chemical properties. Thus, when metabolic dysfunction occurs as a result of inherited mutations in leucine catabolic enzymes, or genes that affect aerobic respiration [1,5], *trans*-3MGC CoA is redirected to form an organic acid waste product (3MGC acid) or it acylates substrate proteins. Based on these findings, it is conceivable that characterization of tissue homogenates obtained from animal models or subjects with primary or secondary 3MGC aciduria will reveal the extent and diversity of protein 3MGCylation in these disorders.

## Data Availability

The data presented in this study are available within the article.
